# Solar Panel Installation Workers Exposed to Pesticides During Two Agricultural Applications — Michigan, August 2023 and May 2024

**DOI:** 10.15585/mmwr.mm7428a2

**Published:** 2025-07-31

**Authors:** Hailey TenHarmsel, Laurel Harduar Morano, Kenneth D. Rosenman

**Affiliations:** 1 Division of Occupational and Environmental Medicine, Michigan State University, East Lansing, Michigan.

**Summary**

**What is already known about this topic?**

Nonagricultural personnel working on or adjacent to farmland can be exposed to direct spray or off-target spray drift from farm-related pesticide applications.

**What is added by this report?**

In August 2023 and May 2024, Michigan’s pesticide surveillance program identified two events involving 10 total solar panel installation workers who required medical evaluation after exposure to pesticides. One worker developed new-onset asthma; all others had no long-term sequelae.

**What are the implications for public health practice?**

To reduce the risk of pesticide exposure among nonagricultural employees working near farmland, employers can notify neighboring farms of their work and coordinate appropriate safety precautions. Persons applying pesticides should follow all product labeling. Local poison control centers can provide guidance regarding acute exposures.

## Abstract

Persons who work near farmland are at risk for exposure to pesticides applied on adjoining agricultural areas. Michigan regulations allow solar panel placement on farmland and open areas near farmland. Nonagricultural workers, including construction workers installing or maintaining solar panels, working in open areas on or adjacent to farmland might be exposed to pesticides yet have little knowledge of the possible health effects. Reports to Michigan’s state pesticide surveillance program from hospitals, emergency departments, the state’s poison center, and emergency service companies identified two separate events, the first in August 2023 and the second in May 2024, when workers installing solar panels experienced illness after pesticide exposures. In these two events, a total of 10 solar panel installation workers reported symptoms temporally related to nearby agriculture pesticide applications. Pesticide applicator adherence to product label instructions is critical to preventing bystander exposure. Increasing awareness of the potential for pesticide exposure among nonagricultural workers near farmland might reduce risk. Employers of nonagricultural workers who are working in rural areas should be aware of agricultural activity surrounding their worksites and should consider contacting farmers to determine pesticide application schedules so that nonagricultural workers can be advised to avoid the area or wear protective equipment during application times. Local poison control centers can provide guidance on management of acute exposures. 

## Introduction

In Michigan, approximately 9.7 million acres (26.6%) of the state’s land are devoted to farming (*1*), and a majority of the farming involves pesticide application (*2*). Michigan regulations allow solar panel placement on farmland (*3*) and open areas near farmland. In 2023, Michigan’s net electricity generation from utility-scale solar power increased by 53% (*4*). Persons who perform any work on or near farmland, including those who install solar panels, are at risk for exposure to agricultural pesticides (*5*).

The approximately 25,000 persons licensed to apply pesticides in Michigan are required to pass an initial examination and obtain continuing educational credits (at least eight and ≥16 for commercial and private licensees, respectively) every 3 years (*6*). Pesticide certification licensing is not required for persons applying pesticides to their own or their employer’s property, provided the pesticide is not classified as a restricted-use product* by the Environmental Protection Agency (EPA). Worker pesticide exposures are investigated by the Michigan Occupational Safety and Health Administration (MIOSHA), which regulates workplace health and safety, and the Michigan Department of Agricultural and Rural Development (MDARD), which has EPA-delegated regulatory authority for pesticides, including pesticide safety for applicators and farmworkers. This report describes two separate incidents, the first in August 2023 and the second in May 2024, when a total of 10 workers installing solar panels adjacent to agricultural land experienced illness after being exposed to pesticides.

## Methods

### Data Sources

An occupational pesticide exposure case is defined as an illness in a worker exposed to a known pesticide who experiences two or more signs or symptoms temporally related to the exposure in a manner plausible or consistent with the product’s known toxicologic effects (*7*). Michigan Public Health Code requires Michigan medical providers, including the Michigan Poison and Drug Information Center^†^ (poison center), emergency medical services (EMS), physicians, hospitals, and emergency departments (EDs) to report cases of pesticide exposure to the state’s pesticide surveillance program. Michigan’s acute pesticide–related illness and injury surveillance program is operated by the Division of Occupational and Environmental Medicine at Michigan State University as a bona fide agent of Michigan’s Department of Health and Human Services. The goal of the program is to track acute pesticide exposures that occurred both at work and at home to help prevent future illness and injury.

### Analysis

Workers’ demographic characteristics, pesticides to which they were exposed, signs and symptoms, and clinical outcomes were evaluated and are described. This investigation was determined by both the Michigan Department of Public Health and the Michigan State University Institutional Review Board (IRB) to be nonresearch public health surveillance and therefore did not require IRB approval.

## Results

### Pesticide Exposure Event A: Miravis Neo Fungicide and Tombstone Insecticide, August 2023

**Exposed workers.** Event A occurred in August 2023 when eight men aged 20–69 years who were installing solar panels became symptomatic immediately after exposure to spray drift from the aerial application of a nonrestricted-use fungicide (Miravis Neo^§^; EPA registration number 100–1605) and a restricted-use insecticide (Tombstone^¶^; EPA registration number 34704–912). The exposure followed the aerial application to crops in a neighboring field by a licensed applicator. The products were applied from an airplane, an estimated 12–50 yards (11–46 m) from approximately 20–25 workers installing solar panels.

Seven of the eight cases were reported to the pesticide surveillance program by the state poison center (Table), including four cases for which the solar panel installation company’s safety manager called the poison center, and three cases for which ED physicians called the poison center. Three cases were reported by hospital EDs and EMS companies, including two that had also been identified by the poison center. Hospital records were requested and reviewed by Michigan’s acute pesticide surveillance program for the six cases that were not reported directly by the hospital. An occupational medicine physician reviewed the medical records. The solar panel installation company’s safety manager reported the names of the pesticides to the poison center after obtaining them from the pesticide application manager, and MDARD reviewed the pesticide application records and confirmed the names of the pesticides. Six of the eight workers agreed to be interviewed by the Michigan acute pesticide surveillance program.

**TABLE:** Characteristics of solar panel installation workers (N = 10) who sought medical care after being exposed to agricultural pesticides during two events — Michigan, August 2023 and May 2024

Characteristic	Exposure event, no. (%)	
	Event A: Miravis Neo* fungicide and Tombstone insecticide^† ^(n = 8) August 2023	**Event B: Roundup PowerMAX 3 herbicide^§^ (n = 2) May 2024**
**Reporting source**		
Poison center only	5 (63)	0 (—)
ED only	1 (13)	0 (—)
Poison center and ED	1 (13)	2 (100)
Poison center and EMS	1 (13)	0 (—)
**Estimated exposed workers**	20–25 (100)	8 (100)
**Workers reporting illness**	8 (32–40)	2 (25)
**Male sex**	8 (100)	2 (100)
**Age group, yrs**		
20–29	3 (38)	0 (—)
30–39	2 (25)	1 (50)
40–49	0 (—)	1 (50)
50–59	2 (25)	0 (—)
60–69	1 (13)	0 (—)
**Reported sign or symptom**		
Cough	6 (75)	1 (50)
Dizziness	5 (63)	0 (—)
Headache	5 (63)	2 (100)
Shortness of breath	5 (63)	0 (—)
Irritated or watery eyes	3 (38)	2 (100)
Nausea	3 (38)	0 (—)
Skin rash, redness, or irritation	3 (38)	2 (100)
Mouth numbness	2 (25)	0 (—)
Muscle weakness	2 (25)	1 (50)
Chest pain	1 (13)	1 (50)
Sore throat	0 (—)	2 (100)
**Hospitalized**	0 (—)	0 (—)

**Abbreviations:** ED = emergency department; EMS = emergency medical services.

* Syngenta US | Miravis Neo | Safety Data Sheet

^†^ Loveland Products | Tombstone | Safety Data Sheet

^§^ Bayer Crop Science United States | Roundup PowerMAX 3 herbicide | Safety Data Sheet

**Symptoms and medical evaluation.** The company’s safety manager telephoned the poison center for medical advice 10 minutes after the exposure. The workers reported dizziness, nausea, cough, irritated eyes, mouth numbness, headache, shortness of breath, chest tightness, skin redness, rash, or irritation, and muscle weakness. All eight workers were evaluated in an ED on the day of the exposure, placed in decontamination showers, and given intravenous fluids; laboratory bloodwork, chest radiographs, and electrocardiograms were analyzed. All workers were discharged from the ED the same day with no additional treatment. One worker who experienced persistent difficulty breathing and coughing at work visited the ED again 7 days later where he received an anticholinergic through a nebulizer and was prescribed a long-acting anticholinergic inhaler and an oral steroid. Four workers were referred by their employer to an occupational medicine physician 1 month after the exposure for ongoing shortness of breath, coughing, weekly headaches, and a sore throat. Symptoms among the other four workers resolved by the time they were discharged from the ED. The workers’ signs and symptoms of eye, skin, and respiratory irritation were consistent with the known toxic effects of the pesticides identified by the manufacturers.** With the exception of one worker who continued to require treatment for new-onset asthma 5 weeks after exposure, all exposed workers’ symptoms were resolved by 5 weeks following the exposure. ED records of the two workers not interviewed indicated their symptoms subsided before they were discharged from the ED.

### Response to Event A Pesticide Exposure

MDARD issued a citation with no monetary penalty to the applicator, stating that the Miravis Neo product had been used in a manner inconsistent with the label, which stated, “[D]o not apply at wind speeds below 3 miles per hour (mph).” Both high and low wind speeds can increase spray drift onto nontarget areas. High wind speeds, generally those above 10 mph (16.1 kph)^††^ increase the spread of the application, whereas low wind speeds (<3 mph) increase potential for variable or gusting winds and can result in temperature inversions, which can cause wider dispersion of the pesticide in cold air trapped beneath warmer air. The wind speed at the time of application was recorded by the applicator as 2 mph. MIOSHA did not receive a complaint.

### Pesticide Exposure Event B: Roundup PowerMAX 3 Herbicide, May 2024

**Exposed workers.** Event B occurred in May 2024 when an estimated eight workers installing solar panels were exposed to nonrestricted-use Roundup PowerMAX 3 herbicide^§§^ (EPA registration number 524–659) that was sprayed from a tractor an estimated 5 feet (1.5 m) from the workers. Neither the solar panel installation company nor their workers were informed of the planned application before it occurred. The workers were told the name of the pesticide by their employer. It is unknown how the employer learned the name of the pesticide.

**Symptoms and medical evaluation**. Two male solar panel installation workers, aged 30 and 49 years, reported sore throat, irritated and watery eyes, skin rash or redness, cough, chest pain, muscle weakness, and headache within hours of exposure (Table). One worker was evaluated in an ED on the day of exposure, was placed in a decontamination shower, and was discharged within 2 hours after his symptoms improved with no additional treatment. The second worker was evaluated in the ED after 2 days of symptoms and released and evaluated again 4 days after the exposure for persistent muscle weakness. In the ED, laboratory bloodwork was analyzed, and he was given a nonsteroidal anti-inflammatory drug for muscle pain with improvement in symptoms. Symptoms of eye, skin, and respiratory irritation reported by the exposed workers were consistent with known toxic effects of the pesticide identified by the manufacturer.^¶¶^ The two exposures were reported to the state surveillance program by the poison center after calls from the treating health care providers at two EDs. Telephone interviews were conducted by the Michigan acute pesticide surveillance program with both exposed workers, who reported the name of the pesticide to the ED physicians and in telephone interviews.

### Response to Event B Pesticide Exposure

MIOSHA issued a citation to the installation workers’ employer for the violation of not ensuring that a safety data sheet for the pesticide was made readily available to the workers within 5 days after the exposure. MIOSHA does not regulate pesticide usage and did not address that the product label specified a 4-hour restricted entry interval, during which persons should not be in the application area. MDARD did not receive a complaint.

## Discussion

In Michigan, approximately 16,000 pesticide products are registered for sale and use (*8*). Pesticide applicator licensing is intended to ensure that pesticide use minimizes environmental hazards and toxicity to applicators and bystanders. Exposure to pesticides can be alarming for persons who do not know what substance they were exposed to and are experiencing symptoms or have concerns about long-term health effects.

Bystanders can be exposed to restricted- and nonrestricted-use pesticides administered by licensed and nonlicensed applicators. Restricted-use pesticides have a higher potential to cause harm and are only available for purchase by persons licensed to use them. All pesticide applicators, irrespective of licensure status, must follow product label instructions and understand how to prevent off-target spray drift, a common cause of pesticide exposure contributing to acute illness (*5*). Identifying the specific pesticide product and contacting a local poison center for treatment advice assists physicians and health care providers who treat workers after an acute pesticide exposure.

Because solar panel installation and other types of nonagricultural work (e.g., county road maintenance) can occur adjacent to farmland, outreach to and education of employers, employees, and health care professionals are needed to ensure that these persons are aware of signs and symptoms of pesticide exposure. Pesticide regulating agencies and occupational health agencies perform different roles in investigating occupational exposure to pesticides. Pesticide regulating agencies such as MDARD review records and determine applicator procedures, even if the applicator is not the employer of who was exposed or reported the exposure. If the complaint is filed promptly, vegetation or clothing samples can be collected in nontarget areas and analyzed for pesticide residue to ascertain whether the spray drifted. Because proper medical management requires knowing the pesticide involved, the pesticide regulatory agency can also identify and notify exposed persons of the involved products. An investigation by the Occupational Safety and Health Administration (OSHA) or a state OSHA program such as MIOSHA will address workplace health and safety. This includes identifying whether workers such as the solar panel installation workers have the ability to determine which products they might be exposed to and whether their employer evaluated engineering and administrative controls to prevent exposure, including access to personal protective equipment. The pesticide regulatory agency investigation is not limited to safeguarding employees and covers independent contractors, owners, and nonworking persons, including children. Pesticide regulating agencies, occupational health programs, and relevant public health authorities can work together to investigate occupational pesticide exposures and provide recommendations, outreach, and education to prevent additional exposures.

### Limitations

The findings in this report are subject to at least four limitations. First, knowledge of events involving nonagricultural workers’ exposure to farmland-applied pesticides is limited by medical provider compliance with state reporting requirements. In event A, six of the eight workers examined in an ED were not reported by the ED as required by Michigan regulations. Second, additional events might have occurred that were not reported because the exposed workers either did not experience symptoms or did not seek medical attention. Therefore, some exposure events might not have been identified in the state’s pesticide surveillance program. Third, the surveillance program is limited to the acute effects and does not address the potential of long-term chronic effects from lower pesticide exposures that do not cause acute symptoms. Finally, these results are limited to a single state and might not be generalizable to other states, although a previous report described bystander exposure in 10 other states (*5*).

### Implications for Public Health Practice

To minimize the risk for pesticide exposure among pesticide applicators and bystanders, all applicators should read, understand, and follow all product labeling. Whether the type of application and product droplet size require an application exclusion zone defined by the EPA Worker Protection Standard should be ascertained by the applicator (*9*). In addition, ensuring adequate distance between the borders of the application and nontarget areas can help avoid spray drift and might be required by the product label. Posted application restrictions, such as a restricted entry interval that does not permit persons in the application area for specific period after application, might also apply.

Employers who work in rural areas should be aware of the farmland surrounding each project site. Because farmers are not required to apprise nonagricultural workers about pesticide application, employers of nonagricultural workers working in rural areas should be aware of agricultural activity surrounding their worksites and should consider contacting farmers to determine pesticide application schedules so that nonagricultural workers can be advised to avoid the area or wear protective equipment during application times.

## Footnotes

*
United States Environmental Protection Agency | Pesticide Worker Safety | Restricted Use Products Report

†
Michigan Poison & Drug Information Center | Wayne State University

§
Syngenta | Miravis Neo fungicide | Product Label

¶
Loveland Products | Tombstone insecticide | Product Label

** Syngenta | Miravis Neo | Safety Data Sheet; Loveland Products | Tombstone | Safety Data Sheet

†† Suggested wind speeds during application will vary by product and required application wind speeds will be stated on the product label.

§§
Roundup PowerMAX 3 herbicide | Product Label

¶¶
Bayer | Roundup PowerMAX 3 herbicide | Safety Data Sheet
